# Loss of SDC1 Expression Is Associated with Poor Prognosis of Colorectal Cancer Patients in Northern China

**DOI:** 10.1155/2019/3768708

**Published:** 2019-04-30

**Authors:** Kaizhi Li, Lei Li, Xiaoxiao Wu, Juan Yu, Hongjun Ma, Renya Zhang, Yan Li, Wei Wang

**Affiliations:** Department of Pathology, Affiliated Hospital of Jining Medical University, Jining Medical University, Jining, Shandong 272029, China

## Abstract

**Background:**

Syndecan-1 (SDC1/CD138) is a key cell surface adhesion molecule essential for maintaining cell morphology and the interactions with the surrounding microenvironment. SDC1 tumor immunoexpression may be increased or decreased in epithelial malignant neoplasms compared to that in adjacent non-neoplastic tissue, depending on the type of carcinoma, and it has been correlated with various clinicopathological parameters and patient prognosis. SDC1 expression is decreased in colorectal cancer (CRC) tissue, but the relationship between prognosis and SDC1 expression in CRC patients is controversial.

**Methods:**

In this study, SDC1 expression was detected in 65 adjacent non-neoplastic colorectal tissues, 477 CRCs, and 79 metastatic lymph nodes using tissue microarray.

**Results:**

The data show that SDC1 decreased in CRC tissues (*p* ≤ 0.001) and metastatic lymph node tissues (*p* ≤ 0.001) compared to that in adjacent non-neoplastic colorectal tissues. Loss of SDC1 protein expression is associated with poor overall (*p* < 0.0001) and disease-free survival (*p* < 0.0001), differentiation (*p* = 0.017), stage (*p* ≤ 0.001), and lymph node metastasis (*p* ≤ 0.001) in CRC patients.

**Conclusions:**

These data suggest that the loss of SDC1 plays an important role in CRC malignant progression. Loss of SDC1 expression indicates poor prognosis in patients from northern China with CRC.

## 1. Introduction

Colorectal cancer (CRC) is the most common tumor of the gastrointestinal system and ranks as the fourth leading cause of cancer-related deaths [1]. The highest incidence rates of CRC are observed in Europe, North America, and Oceania; the lowest rates are reported in Asia, Africa, and South America [2].

SDC1 (syndecan-1, CD138), an important cell adhesion molecule, belongs to the family of syndecans, which are transmembrane heparan sulfate proteoglycans (HSPG) [3]. SDC1 is expressed predominantly in epithelial cells, but it is also found in fibroblasts, myoblasts, and differentiating B cells [4–6]. SDC1 can be cleaved and thus releases the extracellular (ectodomain) core protein-shed SDC1 [7]. The shed SDC1 is increased in response to growth factors, chemokines, heparanase, microbial toxins, insulin, and cellular stress [8, 9]. Although the high shed SDC1 levels in serum have been associated with poor prognosis of CRC patients [10], the relationship between prognosis and epithelial SDC1 expression levels in CRC is controversial [4–6].

In this study, SDC1 expression was detected in 65 adjacent non-neoplastic colorectal tissues, 477 CRC tissues, and 79 metastatic lymph node tissues. The aim of this study was to evaluate the relationship between SDC1 expression and the prognosis of CRC patients from China.

## 2. Materials and Methods

### 2.1. Colorectal Biopsy Specimens

A cohort of 477 (477/621, 76.8%) subjects with CRC, 65 (65/621, 10.5%) adjacent non-neoplastic colorectal epithelia control subjects, and 79 (79/621, 12.7%) subjects with metastatic lymph nodes were recruited between 2008 and 2014 from the Department of Gastrointestinal Surgery in the Affiliated Hospital of Jining Medical University (Shandong, PR China). Of the 477 CRC patients, 250 (52.4%) were male and 227 (47.6%) were female (with a mean age of 61 years). All biopsies were immediately fixed in 4% buffered paraformaldehyde and were routinely processed. Tumors were classified according to the standard TNM staging guidelines of UICC (TNM Classification of Malignant Tumours Eighth Edition). All patients had long-term follow-up results. A cohort of 8 fresh CRC biopsies and paired, adjacent non-neoplastic colorectal tissue samples were collected from patients from the Affiliated Hospital of Jining Medical University. The study protocol was reviewed and approved by the local ethics committee. All patients gave written consent for the tissue samples.

### 2.2. TMA Construction

Representative areas of the CRC, adjacent non-neoplastic colorectal epithelia, and metastatic lymph node tissues were marked on each hematoxylin-eosin (H&E) slide. The TMAs were assembled with a tissue-arraying instrument (Beecher Instruments, Silver Springs, MD, USA) as described by Kallioniemi et al. [11].

### 2.3. Immunohistochemical Staining

Immunohistochemical staining of the SDC1 protein was performed on the TMA slides using the streptavidin-peroxidase (S-P) method as previously described [12]. Briefly, each TMA section was deparaffinized and rehydrated. Antigen retrieval was performed at 95°C in 1x EDTA (ethylenediaminetetraacetic acid) buffer (pH 9.0) for 15 min. Inactivation of endogenous peroxidase was performed by using 0.3% H_2_O_2_-methanol for 30 min. Nonspecific binding was prevented by incubation with normal serum for 20 min at room temperature (RT), followed by incubation with the primary monoclonal antibody against human SDC1 (dilution 1 : 100, Clone No. MI15, Fuzhou Maixin Biotech. Co. Ltd., China) at 4°C overnight. Antibody binding was detected using EnVision reagents (Dako REAL EnVision Detection System; peroxidase/DAB1, DakoCytomation, Denmark). The immune reaction was visualized by incubation with 3,30-diaminobenzidine chromogen substrate (DAB1 Chromogen, DAKOVR, Carpinteria, CA, USA) for 10 min at RT. Finally, slides were counterstained with hematoxylin-eosin, dehydrated, and coverslipped with a mounting automat (Sakura GLC 550, Tissue-TekVR, Alphen aan den Rijn, The Netherlands). SDC1 expression was scored by two independent pathologists without prior knowledge of patients' clinicopathological characteristics. Three nonmetastatic lymph nodes were used as negative (T and B cells) and positive (plasmocytes) controls for SDC1 staining. The color photomicrographs were taken with an upright metallurgical microscope. In tumors (adjacent non-neoplastic glandular epithelium and metastatic lymph nodes), immunohistochemical reactions were classified for intensity as previously described [13, 14]. Briefly, low expression (L, - or ±), no staining (-), weak staining (±), or strong staining was observed in less than 25% of tumor cells. Moderate expression (M, +), moderate staining, or strong staining was observed in only 25-75% of tumor cells. High expression (H, ++) and strong staining were observed in more than 75% of tumor cells.

### 2.4. Protein Extraction and Western Blot

Fresh CRC samples and paired, adjacent non-neoplastic colorectal tissues were homogenized in RIPA lysis buffer (Solarbio, Beijing, China) containing phenylmethylsulfonyl fluoride (PMSF) (Sigma-Aldrich Corporation, St Louis, MO, USA). Equal amounts of protein samples were separated by sodium dodecyl sulfate polyacrylamide gel electrophoresis (SDS-PAGE) and were transferred onto a PVDF membrane (Millipore, Billerica, MA, USA). The membranes were immunoblotted with the following antibodies: monoclonal anti-SDC1 (dilution 1 : 1000, Clone No. 4H5H5) and anti-GAPDH antibodies (dilution 1 : 3000, Proteintech Group Inc., Chicago, IL, USA). The immunoreaction was visualized with enhanced chemiluminescence solution (Millipore, Billerica, MA, USA).

### 2.5. RNA Isolation and Quantitative Real-Time PCR (qRT-PCR)

Total RNA was isolated from fresh CRC samples and paired, adjacent non-neoplastic colorectal tissues using TRIzol Reagent (Invitrogen, San Diego, CA, USA) and then treated with DNase (Roche Diagnostics, Rotkreuz, Switzerland) to eliminate contaminating DNA. Next, 1 *μ*g of the total RNA sample was reverse-transcribed into cDNA using M-MLV reverse transcriptase according to the manufacturer's instructions (Promega, Madison, WI, USA). qRT-PCR was performed using a Bio-Rad iQ SYBR Green Supermix kit and the Bio-Rad iCycler iQ system (Bio-Rad, Hercules, CA, USA). Human SDC1 primers were used with the forward sequence (5′-3′) TGGGGATGACTCTGACAACT and the reverse sequence (5′-3′) CACTTCTGGCAGGACTACAG. Human GAPDH primers were used with the forward sequence (5′-3′) AACGGATTTGGTCGTATTGG and the reverse sequence (5′-3′) TTGATTTTGGAGGGATCTCG. The expression levels of amplified genes were normalized to GAPDH and were presented as relative expression levels.

### 2.6. Statistical Analysis

Pearson's *χ*^2^ test was used to analyze the association between SDC1 expression and clinicopathological characteristics by using the SPSS 13.0 software package (SPSS, Chicago, IL). The Kaplan-Meier method was used to determine the probability of survival, and GraphPad Prism software (version 6, La Jolla, CA, USA) was used to analyze the data with the log-rank test. Differences in quantitative variables between groups were analyzed by Student's *t*-test and Mann-Whitney test (nonparametric text, data do not assume Gaussian distributions). In the analyses, a *p* value of <0.05 was considered significant.

## 3. Results

### 3.1. SDC1 Expression Decreased in CRCs and Metastatic Lymph Nodes

**Figure 1:**
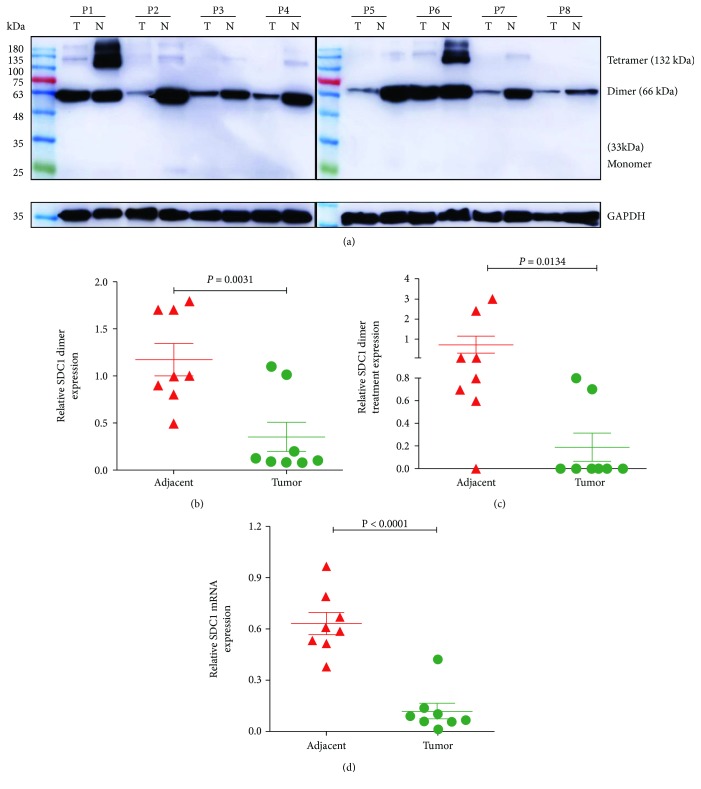
SDC1 expression is decreased in CRCs. (a) Protein expression levels were evaluated in colorectal biopsies taken from 8 tumors (CRCs, T) and adjacent non-neoplastic controls (N) by Western blot. GAPDH was used as a loading control. Quantitative analysis of SDC1 protein: (b) dimer level (*p* = 0.0031, Student's *t*-test) and (c) tetramer level (*p* < 0.0001, Mann-Whitney test) in CRC and paired, adjacent non-neoplastic controls. (d) mRNA expression levels were evaluated in colorectal biopsies and adjacent non-neoplastic controls by qRT-PCR. GAPDH was used as a control. *p* < 0.0001, Student's *t*-test.

We measured SDC1 protein levels by Western blot and mRNA levels by qRT-PCR in 8 fresh CRC samples and paired, adjacent non-neoplastic colorectal tissues. As shown in Figure 1, three expression forms of SDC1 protein were detected, SDC1 protein dimer expression was high (the main expression form), tetramer expression was weak, and monomer expression was absent in all the samples (Figure 1(a)). SDC1 protein was highly expressed in adjacent non-neoplastic colorectal tissue homogenates but was weak or undetectable in CRC tissues (Figure 1(b), dimer, *p* = 0.0031; Figure 1(c), tetramer, *p* = 0.0134); SDC1 mRNA expression was decreased in CRC tissues compared to that in paired, adjacent non-neoplastic colorectal tissues (Figure 1(d), *p* < 0.001).

**Figure 2:**
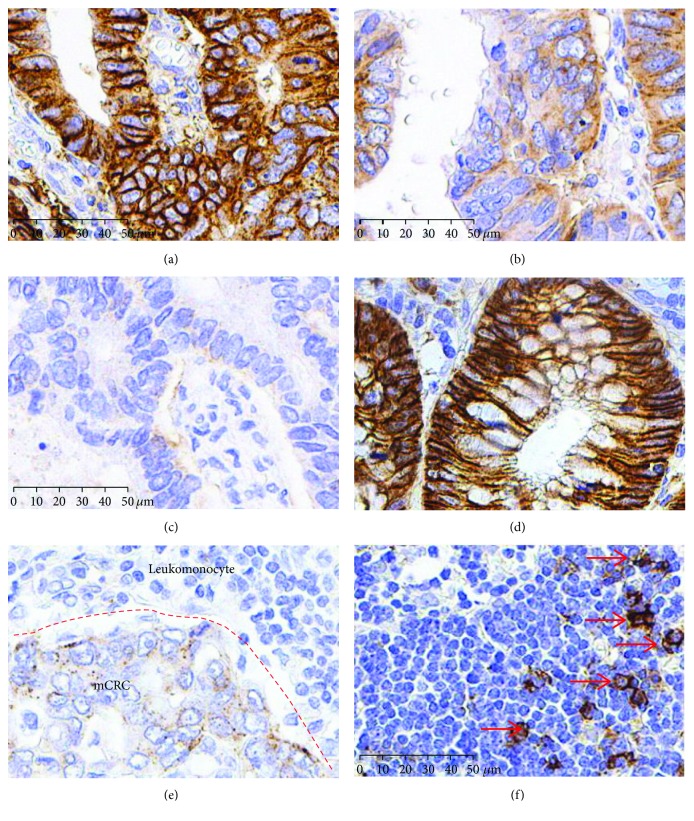
SDC1 decreased both in CRCs and metastatic lymph nodes (mCRC). Immunohistochemical staining for SDC1 in CRCs. (a) High expression, (b) moderate expression, (c) low expression, (d) adjacent non-neoplastic colonic epithelium (high expression), and (e) metastatic lymph nodes (low expression). (f) SDC1 staining in nonmetastatic lymph node as negative control (nonstaining cells, T and B cells) and positive control (red arrow, plasmocytes). Scale bars: 50 *μ*m.

Furthermore, we detected SDC1 expression in 621 cases: 477 samples of CRCs (Figure 2(a), high expression; 2(b), moderate expression; and 2(c), low expression), 65 samples of adjacent non-neoplastic colorectal epithelium (Figure 2(d)), and 79 samples of metastatic lymph nodes (Figure 2(e)) from CRCs by immunohistochemistry (IHC). IHC revealed that high SDC1 expression in adjacent non-neoplastic colorectal epithelial cells was detected mainly in the membrane and cytoplasm (Figure 2(d)). In contrast, SDC1 expression was reduced or undetectable in CRC tissues and metastatic lymph nodes from CRCs. Statistically, among the 65 adjacent non-neoplastic colorectal epithelium samples, 57 (88%) samples showed high expression of SDC1, and 8 (12%) samples showed moderate expression of SDC1. Only 109 of 477 (22.8%) tissues and 228 of 477 (44.8%) CRC samples exhibited high or moderate SDC1 expression, respectively. The immunointensity of SDC1 in metastatic lymph nodes from CRCs further decreased to lower levels since only 11 of 79 (14%) samples showed high SDC1 expression (Table 1). Thus, these data indicate that the loss of SDC1 expression is involved in the development and progression of CRC in northern China.

### 3.2. Association between SDC1 Expression in CRC Tissues and Patients' Clinicopathological Characteristics

According to the SDC1 staining intensity and the extent of positive tumor cells, our data showed that SDC1 was expressed at low levels in 29.4% (140/477) of CRCs, moderately expressed in 44.8% (228/477) of CRCs, and highly expressed in 22.8% (109/477) of CRCs (Table 1). We next assessed the relationship between SDC1 expression and patients' clinicopathological characteristics. SDC1 expression was not correlated with age (*p* = 0.345), sex (*p* = 0.686), or tumor diameter (*p* = 0.232). However, loss of SDC1 protein was significantly associated with poor differentiation (*p* = 0.017), advanced TNM stage (*p* ≤ 0.001), and LN metastasis (*p* ≤ 0.001) (Table 2).

### 3.3. Loss of SDC1 Expression Is Significantly Associated with Poor Prognosis of ESCC Patients

**Figure 3:**
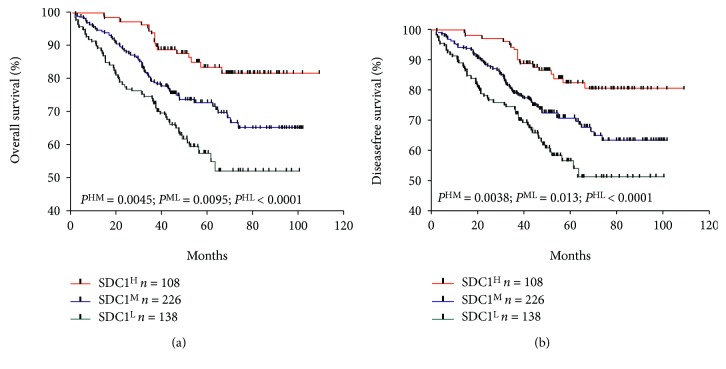
Relationship between CRC SDC1 status and patient survival. The Kaplan-Meier survival curves demonstrate that low, moderate, and high SDC1 expression in the tumors did correlate with (a) overall survival and (b) disease-free survival.

Kaplan-Meier analysis revealed that patients who exhibited reduced SDC1 (moderate or low) expression were associated with poorer overall survival and disease-free survival compared to patients who exhibited high SDC1 expression (*p* = 0.0045, *p* < 0.0001 and *p* = 0.0038, *p* < 0.0001, respectively, Figures 3(a) and 3(b)). These data indicate that SDC1 plays a role as a reliable tumor suppressor in CRC.

## 4. Discussion

Our findings derive from a large, clinically annotated tissue microarray of CRC specimens and add to the body of evidence that the loss of epithelial SDC1 is a general feature of carcinoma progression. The loss of SDC1 expression in local lymph node metastasis is evidence of the prometastasis function of SDC1. In fact, our results showed that the loss of expression of epithelial SDC1 truly correlates with poor dedifferentiation, stage, and local lymph node metastasis in CRC. In agreement with other analyses of CRC, the loss of epithelial SDC1 was correlated with tumor TNM stage [4–6, 13], and the incidence of metastasis was correlated with local lymph nodes [4–6].

SDC3 and SDC4 have been reported as oncogenes [15, 16]. Recently, syndecan-2 (SDC2) methylation was highlighted as a potential marker for early CRC detection. A DNA microarray analysis of neoplastic samples showed a high SDC2 methylation rate of approximately 95%, regardless of the early colorectal cancer stage [17]. Blood SDC2 methylation data from 131 CRC patients and 125 healthy subjects showed a high sensitivity of 92.3% for detecting stage I CRCs [18]. Bowel lavage fluid (BLF) SDC2 methylation data showed that SDC2 methylation was positive in 100% of villous adenoma, high-grade dysplasia, and hyperplastic polyp samples; in 88.9% of tubular adenoma samples; and in 0% of normal mucosal samples [19]. These results suggest that the reduction in SDC1 expression in CRCs may also be caused by *SDC1* DNA methylation, and further research is needed.

The syndecan transmembrane domain and transmembrane domain-induced dimerization seem to critically regulate various functions of syndecan family members [20]. Research has shown that SDC1 is coexpressed with EMT markers (E-cadherin and *β*-catenin) in CRCs and that this coexpression is regulated during epithelial-mesenchymal transition (EMT) [21]. The loss of SDC1 expression in carcinoma cells reduces cell adhesion to the extracellular matrix and enhances cell motility and invasion [22]. Our results showed that SDC1 expression was mainly in the form of a dimer in normal colorectal epithelial cells and was downregulated in CRCs. This suggests that SDC1 is inactivated in CRCs, thus reducing cell adhesion to the extracellular matrix and enhancing cell motility and invasion.

We also found that the loss of expression of epithelial SDC1 significantly correlates with poor patient survival. Previous studies reached conflicting conclusions on whether reduced SDC1 is correlated with decreased patient survival [4–6]. A study from Japan revealed that the low expression of epithelial SDC1 was significantly associated with poor clinical outcome in CRC [11], but two studies from Finland and the USA have shown that the low expression of epithelial SDC1 did not significantly correlate with the survival of CRC [23, 24]. It is important to note that the studies that have examined the use of SDC1 as a prognostic marker were performed in different countries and on different continents [4–6]. Therefore, other factors such as treatment plans, genetic variations, and ethnicity may have influenced the results and affected the prognostic value of SDC1 in CRC progression and metastasis. Our data are in agreement with data from the study of Fujiya et al. [14]. The data suggest that the relationship between SDC1 expression and the prognosis of CRC patients may have ethnic and regional differences: the loss of SDC1 expression was correlated with a poor prognosis for East Asian CRC patients but not for Europeans.

In summary, the loss of SDC1 expression in CRC is closely associated with poor differentiation, stage, and local lymph node metastasis. SDC1 is a valuable biomarker for predicting the prognosis of CRC patients in northern China.

## Figures and Tables

**Table 1:** Loss of SDC1 expression in CRCs and metastatic lymph nodes, 621 cases.

	SDC1^L^ *N* = 180 Case (%)	SDC1^M^ *N* = 264 Case (%)	SDC1^H^ *N* = 177 Case (%)	Total	*χ* ^2^	*p*
Adjacent non-neoplastic tissues^a^	0 (0)	8 (12)	57 (88)	65	114.154	≤0.001^ab^
CRCs^b^	140 (29.4)	228 (47.8)	109 (22.8)	477	151.685	≤0.001^bc^
Metastatic lymph nodes^c^	40 (51)	28 (35)	11 (14)	79	81.639	≤0.001^ac^

CRC: colorectal cancer; L: low expression; M: moderate expression; H: high expression; statistical method: chi-square test.

**Table 2:** Relationship between SDC1 immunoreactivity and clinicopathological characteristics, 477 CRC cases.

Clinical information	Total	SDC1^L^ *N* = 140 Case (%)	SDC1^M^ *N* = 228 Case (%)	SDC1^H^ *N* = 109 Case (%)	*χ* ^2^	*p*
*Age, yr*						
<60.5	211	67 (32)	93 (44)	51 (24)	2.13	0.345
>60.5	266	73 (27)	135 (51)	58 (22)		
*Gender*						
Female	227	69 (30)	110 (49)	48 (21)	0.752	0.686
Male	250	71 (28)	118 (47)	61 (24)		
*Tumor size, cm*						
≤4	306	92 (30)	138 (45)	76 (25)	2.924	0.232
>4	171	48 (28)	90 (53)	33 (19)		
*Histological grade (differentiation) (miss samples,N* = 16)						
Well	226	75 (33)	111 (49)	40 (18)	8.12	0.017
Moderately or poorly	235	59 (25)	110 (47)	66 (28)		
*TNM stage (T)*						
1-2	69	14 (20)	22 (32)	33 (48)	28.55	≤0.001
3-4	408	126 (31)	206 (50)	76 (19)		
*Lymph node metastasis*						
N_0_	290	66 (23)	140 (48)	84 (29)	23.088	≤0.001
N_1-3_	187	74 (40)	88 (47)	25 (13)		
*Location (miss samples,N* = 286)						
Left	49	17 (35)	23 (47)	9 (18)	1.123	0.570
Right	142	38 (27)	74 (52)	30 (21)		

L: low expression; M: moderate expression; H: high expression; statistical method: chi-square test.

## Data Availability

The data used to support the findings of this study are available from the corresponding author upon request.
